# Isolated Rapid Deployment Aortic Valve Replacement in Patients with Aortic Stenosis: Single-Center Retrospective Study

**DOI:** 10.3390/jcdd12050191

**Published:** 2025-05-17

**Authors:** Ricardo Ferreira, Tiago R. Velho, João Gonçalves, André Sena, Beatriz Draiblate, Ana G. Almeida, Ângelo Nobre, Fausto Pinto

**Affiliations:** 1 Department of Cardiothoracic Surgery, Hospital de Santa Maria, Unidade Local de Saúde Santa Maria, 1649-035 Lisbon, Portugal; tiagovelho48@hotmail.com (T.R.V.); joaog@campus.ul.pt (J.G.); ags.neon@gmail.com (A.S.); beatriz.draiblate@ulssm.min-saude.pt (B.D.); angelo.nobre@ulssm.min-saude.pt (Â.N.); 2 Centro Cardiovascular da Universidade de Lisboa (CCUL@RISE), Faculdade de Medicina, Universidade de Lisboa, 1649-028 Lisbon, Portugal; anagalmeida@gmail.com (A.G.A.); fausto.a.pinto@ulssm.min-saude.pt (F.P.); 3 Department of Cardiology, Hospital de Santa Maria, Unidade Local de Saúde Santa Maria, 1649-035 Lisbon, Portugal

**Keywords:** aortic stenosis, rapid deployment bioprosthesis, elderly patients, surgical outcomes

## Abstract

Background: Aortic valve stenosis remains the most prevalent valvular pathology in Western countries. Rapid deployment bioprosthesis (RD) has emerged as a promising alternative to conventional valves for surgical aortic valve replacement (SAVR), particularly in elderly and high-risk patients. This study reports the short- and long-term outcomes of RD in patients with isolated aortic stenosis. Methods: A retrospective single-center analysis was conducted on 382 patients who underwent RD-AVR between 2014 and 2020. Data were collected from clinical files and national electronic databases. Primary outcomes included cardiopulmonary bypass (CPB) and cross-clamping (XC) times, postoperative complications, and long-term survival. Results: The mean age was 75.6 ± 5.9 years, with 29.8% of patients over 80 years old and a mean EuroSCORE II of 2.3 ± 1.5%. CPB and XC times were 36.7 ± 10.8 and 27.4 ± 8.1 min, respectively. Postoperative complications included acute kidney injury (AKI, 53.4%), de novo atrial fibrillation (31.9%), and high-grade/complete atrioventricular block with permanent pacemaker implantation (9.8%). In-hospital and 30-day mortality was 1.02% and 2.3%, respectively. The 5-year survival rate was 77%. At 6 months postoperatively, the mean transvalvular gradient was 11.1 ± 4.7 mmHg. At a median follow-up of 6.7 years, no cases of structural valve deterioration and only one case of endocarditis were reported. Conclusion: In this single-center study, RD in isolated AVR demonstrated favorable short- and long-term outcomes, including no structural valve deterioration at mid-term follow-up. These devices offer a safe and effective alternative to conventional SAVR, particularly in high-risk populations.

## 1. Introduction

Aortic valve stenosis is the most common valvular pathology in Western countries, affecting about 5% of individuals at age 65 and up to 10% in those over 80 years [1].

Elderly patients often present with multiple comorbidities, which have been classically associated with increased morbidity and mortality after cardiac surgery, reinforcing the necessity for improvements in patient selection, perioperative management, surgical techniques, and devices, including strategies to minimize cardiopulmonary bypass (CPB) and cross-clamping (XC) duration and impact [2,3].

In recent years, a new concept of surgical bioprosthetic valves has emerged, known as rapid deployment (RD) aortic devices. These valves incorporate transcatheter technology elements and were developed with the goal of simplifying the implantation process, thereby potentially reducing CPB and aortic XC times and aortic manipulation [4,5].

This feature seems particularly useful in older/high-risk patients, reoperations, and/or combined procedures. Moreover, by avoiding or minimizing the need for annular sutures, they allow expansion to the full size of the aortic annulus, which may contribute to better hemodynamics, especially beneficial for patients with a small aortic annulus; however, comparative short- and long-term results relative to conventional surgical valves remain under investigation, as with transcatheter valves [6,7,8].

The aim of this study was to report our initial single-center experience using RD devices for isolated surgical aortic valve replacement (SAVR) in patients with aortic stenosis.

## 2. Methods

### 2.1. Study Population

This single-center retrospective analysis included 382 consecutive patients diagnosed with isolated severe aortic valve stenosis who underwent surgical aortic valve replacement (SAVR) using an RD (Intuity^®^ [Edwards Lifesciences Corporation, Irvine, CA, USA] or Perceval^®^ [Corcym Group, Milan, Italy]) between January 2015 and December 2020. During the study period (2015–2020), approximately 1018 isolated SAVR procedures were performed at our institution. The present study cohort comprises 37% of the total isolated SAVR volume during this timeframe. Reoperations were not included in this study.

Demographics, clinical characteristics, perioperative and follow-up data were collected electronically from the clinical files from our department and from registries from the national electronic healthcare database.

Postoperative echocardiographic follow-up was typically performed at discharge, 6 months postoperatively, and annually thereafter, according to institutional protocol, or more frequently if clinically indicated.

### 2.2. Surgical Procedure

The indications for SAVR were consistent with the European Society of Cardiology/European Association for Cardio-Thoracic Surgery (ESC/EACTS) Guidelines for the management of valvular heart disease applicable at the time of the interventions [9].

The selection criteria favoring the use of RD valves over conventional SAVR were not rigidly protocolized during this period and often depended significantly on individual surgeon preference. However, RD valves were preferentially considered for patients older than 75 years and in cases presenting anticipated technical challenges, such as the presence of extended annular, aortic, or left ventricular outflow tract calcification; expected difficulty with conventional annular suturing; or documented small aortic annulus diameter.

The surgical approach for all patients was standard median sternotomy, with normothermic cardiopulmonary bypass. The aortotomy was adjusted to the implanted device, and the native valve was completely excised. The bioprosthesis was sized and implanted according to the manufacturer’s instructions to achieve optimal hemodynamic performance and avoid patient-prosthesis mismatch. De-airing was performed routinely with CO_2_.

Valve function and position were evaluated by intraoperative transesophageal echocardiography (TEE) in all patients.

### 2.3. Statistical Analysis

Categorical variables are reported as absolute frequencies and percentages. Continuous variables are presented as mean ± standard deviation (SD) for normally distributed data or median [interquartile range, IQR] for non-normally distributed data. Cumulative survival and freedom from events were estimated using the Kaplan–Meier method, with 95% confidence intervals (CI). All statistical analyses were performed using IBM SPSS Statistics (Version 27, IBM Corp., Armonk, NY, USA) and GraphPad Prism 9 (GraphPad Software, Boston, MA, USA).

## 3. Results

### 3.1. Baseline Characteristics

**Figure 1:**
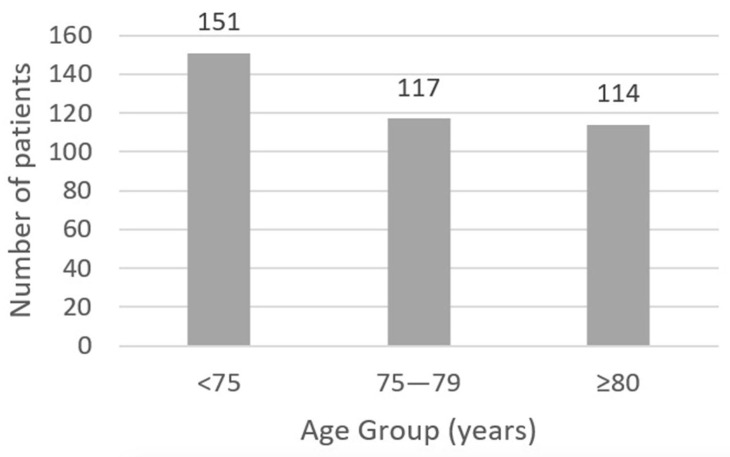
Preoperative age distribution by groups. Patients <75 years (*n* = 151), between 75 and 79 years (*n* = 117), and those over 80 years old (*n* = 114).

Baseline characteristics are detailed in Table 1. The mean overall age was 75.6 ± 5.9 years, with an equal gender distribution (50.0% male). As shown in Figure 1, almost one-third of the study population was over 80 years old (29.8%), and the mean predicted in-hospital mortality risk (EuroSCORE II) was 2.3 ± 1.5%. Prevalent preoperative risk factors included arterial hypertension (87.3%), impaired renal function [eGFR < 90 mL/min/1.73 m^2^] (81.9%), dyslipidemia (76.7%), and diabetes mellitus (38.2%). Atrial fibrillation was present preoperatively in 21.7% of the cohort, and 7.5% had a history of prior stroke or transient ischemic attack. The vast majority of patients (82.1%) had preserved left ventricular (LV) function, defined as an ejection fraction higher than 50%.

### 3.2. Surgery and Postoperative Outcomes

Most patients received an Intuity^®^ valve (*n* = 227, 59.5%). The mean CPB and aortic XC times were 36.7 ± 10.8 min and 27.4 ± 8.1 min, respectively. Apart from four Perceval^®^ valves requiring repositioning due to misplacement identified at intraoperative TEE, no other significant intraoperative complications or relevant paravalvular leaks were reported.

The projected indexed effective orifice area (iEOA) was calculated based on information provided by the manufacturer (minimal effective orifice area possible for each valve size) [10]. No cases of severe Patient Prosthesis Mismatch (PPM), defined as an iEOA ≤ 0.65 cm^2^/m^2^ if BMI < 30 kg/m^2^ or iEOA ≤ 0.55 cm^2^/m^2^ if BMI ≥ 30 kg/m^2^, were identified; however, we identified 20 patients (8.8%) that received an Intuity^®^ device with moderate PPM (iEOA ≤ 0.85 cm^2^/m^2^ if BMI < 30 kg/m^2^ or iEOA ≤ 0.70 cm^2^/m^2^ if BMI ≥ 30 kg/m^2^) [11].

Postoperative outcomes are described in Table 2. The mean average intensive care unit (ICU) stay was 3.0 ± 2.5 days (median 3 days—IQR 2–4), and the mean overall hospital stay was 6.6 ± 3.8 days (median 6 days—IQR 4–8). Common postoperative complications included the need for prolonged (>24 h) or high-dose inotropic support (24.3%), acute kidney injury (AKI any stage, 53.4%), and de novo atrial fibrillation (*n* = 92/199, 31.9%). High-grade or complete atrioventricular block requiring definitive pacemaker implantation occurred in 9.8% of patients (*n* = 36/367). Most patients had no significant postoperative bleeding (85.1%), but 2.8% (*n* = 11) required surgical re-exploration for bleeding or tamponade. The stroke rate was low at 0.5% (*n* = 2). In-hospital mortality and 30-day mortality were 1.02% (*n* = 4) and 2.3% (*n* = 9), respectively.

### 3.3. Follow-Up

**Figure 2:**
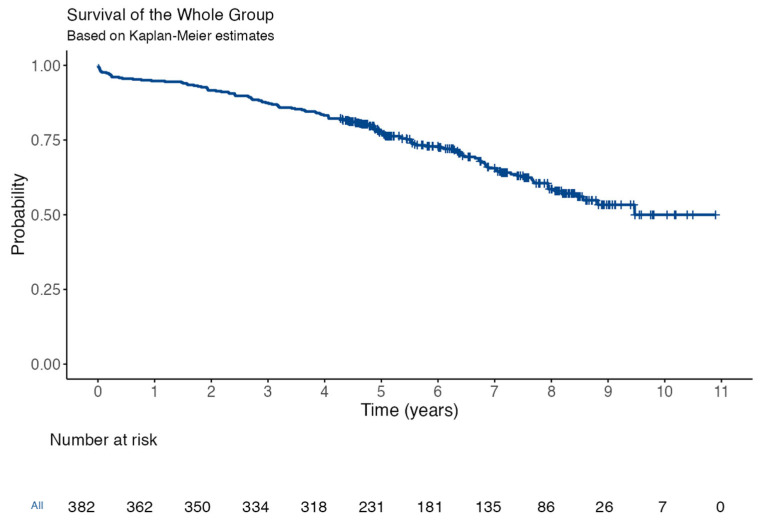
Follow-up overall mortality (Kaplan–Meier overall survival curve—95% confidence interval).

During a mean follow-up of 6.7 ± 1.8 years, no cases of structural valve deterioration were reported according to the Valve Academic Research Consortium 3 criteria [12]. One case of prosthetic endocarditis occurred, requiring antibiotic therapy and reoperation. Mean transvalvular gradients assessed by TTE at 6 months or later were 11.1 ± 4.7 mmHg. The Kaplan–Meier estimated an overall 1-year and 5-year cumulative survival rate of 94.4 ± 1% and 77 ± 2%, respectively (Figure 2).

## 4. Discussion

It is well established that aortic XC and CPB times are independent predictors of morbidity and mortality in cardiac surgery [13]. Consequently, significant efforts have been made to minimize these factors.

One such development is the creation of RD devices, a hybrid technology combining surgical and percutaneous concepts. These devices allow for implantation with reduced (e.g., Intuity^®^) or no (e.g., Perceval^®^) anchoring sutures, offering the potential to substantially reduce surgical times, irrespective of surgeon experience [8,14].

Recognizing the potential impact of these devices, particularly in older/high-risk patients or those undergoing combined procedures, several surgical centers began utilizing them, contributing to a growing body of evidence on their outcomes [15].

Despite direct comparisons with specific trials that should be made carefully, our reported mean CPB and XC times (36.7 and 27.4 min, respectively) for isolated RD-AVR are comparable to or shorter than those reported in several large series and meta-analyses of RD valves [15,16]. There were no major intraoperative complications apart from the need for Perceval^®^ bioprosthesis repositioning in four patients, a known technical aspect manageable with established techniques [17].

Our study demonstrates favorable short- and mid-term results in this cohort, where nearly one-third of patients were over 80 years old. The observed in-hospital mortality (1.02%) and 30-day mortality (2.3%) were low and compared favorably to the predicted risk based on EuroSCORE II [18]. Stroke rates were also low (0.5%). Although over 50% of patients experienced some degree of AKI postoperatively (a common finding after CPB), severe AKI requiring renal replacement therapy was infrequent (2.3%) [19,20].

The relatively high rate of postoperative inotropic support (24.3%) might be related, in part, to specific institutional fluid management protocols used in our department but warrants consideration. New-onset atrial fibrillation occurred in 31.9%, a rate consistent with the known multifactorial etiology and high incidence of this arrhythmia following any cardiac surgery [21].

The overall incidence of permanent pacemaker implantation (PPI) was 9.8%. This rate is within the range described in the literature for RD devices, which is generally higher than that observed with conventional stented bioprosthesis [22,23]. Current evidence suggests that baseline conduction system disturbances (particularly right bundle branch block) and prosthesis oversizing are significant predictors of PPI with RD valves, which can negatively impact outcomes [24]. Careful patient selection, precise annular sizing, and standardized implantation techniques (e.g., implantation depth) are crucial to minimize this complication rate. At our institution, pacemaker implantation is typically considered for persistent high-grade/complete AV block beyond postoperative days 5–7 or symptomatic bradyarrhythmia.

Midterm follow-up showed good valve durability, with no structural valve deterioration reported at a median of 6.7 years. The low mean postoperative transvalvular gradients and the absence of severe PPM confirm the favorable hemodynamic performance of these devices. The overall 5-year survival rate of 77% in this cohort, characterized by a mean age over 75 years and significant comorbidities, is encouraging.

Also significant was the fact that only one patient (0.2%) had infectious endocarditis. Some authors mention that because these valves allow for less manipulation of the aortic root/annulus and no or limited permanent contact with foreign material such as sutures, they present higher resistance against endocarditis in comparison with conventional prostheses [25,26].

Our observed low perioperative mortality and stroke rates with RD-AVR appear competitive with contemporary outcomes reported for both SAVR and TAVI, particularly in low- and intermediate-risk cohorts [27]. Notably, the permanent pacemaker implantation in our cohort, while higher than conventional SAVR, falls within the wide range reported for different TAVI valve types and implantation techniques [22]. Furthermore, RD-AVR maintains the surgical advantages of direct annular visualization and complete native valve excision, allowing commissural alignment and minimizing the risk of significant paravalvular leak compared to TAVI [28,29]. Given the advantages of RD-AVR in simplifying the surgical procedure compared to conventional SAVR and the minimally invasive nature of TAVI, future research, including randomized controlled trials, should directly compare RD-AVR with TAVI. Such comparisons are warranted, particularly in low- and intermediate-risk patients or patient subgroups with specific anatomical considerations (e.g., challenging TAVI access, specific annular morphologies), to better delineate the optimal therapeutic strategy for individual patients in the current era [30].

## 5. Limitations

This study has several limitations inherent to its design. It is a retrospective analysis from a single center, lacking a concurrent control group of patients receiving conventional SAVR. This prevents direct comparative conclusions regarding operative times or specific complication rates versus traditional valves within our own institution. Patient selection for RD valves was not randomized and was subject to surgeon preference, potentially introducing selection bias. While data were collected systematically, reliance on electronic records might lead to underreporting of minor events. Follow-up, while complete for mortality via national registries, may be less comprehensive for clinical or echocardiographic details in some patients lost to local follow-up. Future studies, ideally randomized controlled trials comparing RD-AVR with contemporary conventional SAVR prostheses and transcutaneous aortic valve implantation (TAVI), are needed to further define the relative merits and optimal indications for these devices.

## 6. Conclusions

In this single-center retrospective analysis, isolated SAVR using RD was associated with favorable short- and mid-term outcomes in a cohort with a high proportion of elderly patients. Observed mortality was low, and valve durability appeared excellent at mid-term follow-up, with no cases of structural deterioration reported. While associated with a higher rate of pacemaker implantation compared to traditional valves, RD-AVR represents a safe and effective surgical option, particularly valuable in contexts where minimizing operative times is advantageous.

## Figures and Tables

**Table 1:** Preoperative baseline characteristics and risk factors.

*N*	382
Age (years), mean ± SD	75.6 ± 5.9
Male Sex, *n* (%)	190 (50.0)
Euroscore II (%), mean ± SD	2.3 ± 1.5
Diabetes Mellitus (DM), *n* (%)	146 (38.2)
Insulin-Treated DM, *n* (%)	17 (4.4)
Atrial Fibrillation, *n* (%)	83 (21.7)
Previous Pacemaker, *n* (%)	15 (3.9)
Impaired Kidney Function, *n* (%)	313 (81.9)
Hemodialysis, *n* (%)	8 (2.1)
Respiratory Disease *, *n* (%)	84 (21.9)
Smoker|Ex-Smoker, *n* (%)	96 (25.1)|86 (22.5)
Carotid Disease **, *n* (%)	39 (10.2)
Cerebrovascular Disease ***, *n* (%)	29 (7.5)
LVEF > 50%, *n* (%)	314 (82.1)

Impaired Kidney Function—eGFR (estimated Glomerular Filtration Rate) < 90 mL/min/1.73 m^2^—calculated using the CKD-EPI formula. LVEF—left ventricular ejection fraction. * Respiratory Disease—included asthma and Chronic Obstructive Pulmonary Disease (COPD). ** Carotid Artery Stenosis—defined as documented carotid artery stenosis >50%. *** Cerebrovascular Disease—included history of stroke or transient ischemic attack.

**Table 2:** Postoperative outcomes.

Postoperative Outcomes	*N* = 382
Length of Stay	
ICU Stay (days), median [IQR] (mean ± SD)	3 [2–4] (3.0 ± 2.5)
Total Hospital Stay (days), median [IQR] (mean ± SD)	6 [4–8] (6.6 ± 3.8)
Postoperative Complications	
Hemodynamic Support > 24 h †, *n* (%)	93 (24.3)
Acute Renal Injury (AKI, any stage), *n* (%)	204 (53.4)
AKI (AKIN ≥ 2), *n* (%)	35 (9.2)
Renal Replacement Therapy, *n* (%)	9 (2.3)
De Novo Atrial Fibrillation, *n* (%)	92/299 (31.9)
Definitive Pacemaker Implantation, *n* (%)	36/367 (9.8)
Stroke, *n* (%)	2 (0.5)
Seizures, *n* (%)	8 (2.0)
Infection ‡, *n* (%)	26 (6.8)
Significative Bleeding §, *n* (%)	57 (14.9)
Reoperation for Bleeding, *n* (%)	11 (2.8)
Mortality	
In-Hospital Mortality, *n* (%)	4 (1.02)
30-Day Mortality, *n* (%)	9 (2.3)

ICU: intensive care unit; AKI: acute kidney injury (defined by AKIN criteria); IQR: interquartile range; SD: standard deviation. † defined as a requirement for moderate-/high-dose inotropes or vasopressors for >24 h. ‡ defined as sepsis, deep sternal wound infection, pneumonia, or endocarditis requiring prolonged antibiotics or intervention. § defined as bleeding requiring transfusion of >2 units of packed red blood cells or surgical re-exploration.

## Data Availability

The data presented in this study are available on request from the corresponding author.
